# Bile acid mediated effects on gut integrity and performance of early-weaned piglets

**DOI:** 10.1186/s12917-015-0425-6

**Published:** 2015-05-14

**Authors:** Nuria de Diego-Cabero, Alessandro Mereu, David Menoyo, Jens J. Holst, Ignacio R. Ipharraguerre

**Affiliations:** Departamento de Producción Animal, Universidad Politécnica de Madrid, E.T.S. Ingenieros Agrónomos, Ciudad Universitaria S/N, Madrid, 28040 Spain; Lucta S.A., Montornés del Vallés, Barcelona, 08170 Spain; The NNF Center for Basic Metabolic Research and Department of Biomedical Sciences, University of Copenhagen, Copenhagen, DK-2200 Denmark; Institute of Human Nutrition and Food Science, Christian-Albrechts-University, D-24118 Kiel, Germany

**Keywords:** Chenodeoxycholic acid, Glucagon-like petide-2, Early weaning, Piglet

## Abstract

**Background:**

Early weaning (EW) results in a transient period of impaired integrity of the intestinal mucosa that may be associated with reduced plasma concentration of glucagon-like peptide-(GLP) 2. We have previously shown that intragastric infusion of chenodeoxycholic acid (CDC) increases circulating GLP-2 in early-weaned piglets. The aim of this study was to expand previous work to establish whether feeding piglets a cereal-based diet supplemented with CDC can improve gut integrity and animal performance immediately after EW. A cohort of 36 piglets weaned at 20 days of age, 6.2 ± 0.34 kg of body weight (BW) were randomly assigned (*n* = 18) to receive a standard prestarter diet or the same diet supplemented with 60 mg of CDC per kg of initial BW for *ad libitum* intake until day 14 postweaning. Thereafter, all pigs were fed the same untreated starter diet for 21 days until the end of the study on day 35. On days 1, 7 and 14 blood samples were collected from 6 pigs per treatment to measure plasma GLP-2. On day 15, 6 pigs per treatment were euthanized to obtain intestinal tissue samples for later histological and gene expression analyses.

**Results:**

Supplementing the diet with CDC tended to increase plasma GLP-2 (P < 0.07; 39 %) and the weight of the large intestine (P < 0.10; 11 %), and increased ileal crypt depth (P < 0.04; 15 %) after 14 days of treatment exposure. Although feed intake and BW gain were not affected by treatments, feeding CDC induced the expression of the cytokines *TNF-α* (P < 0.02; 1.9 fold*), IL-6* (P < 0.01; 2.4 fold), and *IL-10* (P < 0.006; 2.2 fold) and the tight junctional protein *ZON-1* (P < 0.02; 1.5 fold) in the distal small intestine.

**Conclusions:**

This study showed that the oral administration of CDC to early-weaned pigs has the potential to improve the protection of the intestinal mucosa independently of relevant changes in gut growth.

## Background

Early weaning (EW) is a widespread practice in modern settings of pig production. At that time, piglets are exposed to a variety of stressors including abrupt separation from sow and changes in diet and environment, which jointly result in a period of transient anorexia, gut mucosal atrophy, and intestinal dysfunction [1–3]. The weaning-induced deterioration of gut integrity could be partly related to the marked reduction in circulating glucagon-like peptide (GLP)-2 that typically accompanies EW in pigs [4, 5].

GLP-2 is an intestinotrophic peptide released by the enteroendocrine L cells mainly in response to luminal nutrients [6, 7]. Of interest, exogenous GLP-2 restores mucosal growth, transcellular transport, and the expression of tight junction (TJ) proteins that control paracellular permeability in a number of animal models of intestinal atrophy or dysfunction [8–10]. Recently, it has been found that the chronic administration of GLP-2 at supraphysiological levels to neonatal pigs for 42 days increased villus height and crypt depth in the small intestine and colon 21 days after EW [11]. More important, the administration of a long-acting analog of GLP-2 at pharmacological doses to 25-days-old suckling piglets increased intestinal weight and enzyme activity 5 days after weaning [12]. Although available evidence suggests that GLP-2 treatment can contribute to improve intestinal adaptation to weaning, it is reasonable to expect that strategies capable of enhancing secretion and (or) stability of endogenous GLP-2 might be equally effective but easier to implement under commercial schemes of pig production.

In recent years bile acids have emerged as potent hormonal regulators capable of stimulating the secretion of GLP-1 (a co-product of proglucagon, released in parallel with GLP-2) from the intestine. This action is mediated by the G-protein-coupled bile acid receptor 1 (GPBAR1, also known as TGR5), which is a bile acid sensor expressed on the luminal surface of intestinal L cells [13, 14]. Interestingly, the continuous enteral administration of chenodeoxycholic acid (CDC), a primary bile acid known to activate TGR5, to newborn piglets fed parenterally increased the plasma concentration of GLP-2 and prevented gut atrophy otherwise resulting from the lack of enteral nutrition [15]. In a later study, we investigated whether CDC could induce a similar response in weanling pigs. In this study, piglets weaned at 21 days of age, fed a cereal-based diet, and infused intragastrically with a single dose of CDC had increased circulating GLP-2 and tended to have a longer and heavier intestine than their control counterparts [16]. As proposed in that report, it is plausible that the dose of CDC and administration procedure used in our study might have limited the impact of increased GLP-2 secretion on intestinal adaptation to EW. It is important to note, however, that bile acids may also control the integrity of the intestinal barrier by regulating the expression or cellular distribution of TJ proteins through mechanisms unrelated to GLP-2 [17, 18].

In summary, available evidence indicates that activating intestinal signaling pathways controlled by bile acids allows stimulating the release of endogenous GLP-2 and thereby improving gut integrity in experimental models of intestinal atrophy and dysfunction. Therefore, the aim of this study was to expand previous work to establish whether the inclusion of CDC in the diet of early-weaned piglets fed according to current standards of pig production can improve gut integrity and animal performance immediately after weaning.

## Methods

### Animals and housing

All experimental procedures were approved by the Laboratory Animal Care Advisory Committee of the Faculty of Veterinary Sciences of the Universitat Autónoma de Barcelona, Spain. A total of 36 pigs (Large White x Landrace x Pietrain; 18 of each sex) weaned at 20 ± 0.9 days of age and 6.2 ± 0.34 kg of body weight (BW) were used in a study conducted at the Swine Experimental Unit of Lucta S.A. (Girona, Spain). At arrival, piglets were distributed into 36 individual pens (0.35 m^2^/pen) thoroughly cleaned and equipped with fully-slatted plastic floor plus a nipple drinker and a feeder. Animals were randomly assigned to receive a standard prestarter diet (CONd; *n* = 18; 50:50 male to female ratio) or the same diet supplemented with 60 mg of CDC (Sigma-Aldrich) per kg of initial BW (CDCd). Animals were fed the solid diets from weaning until day 14; thereafter, all pigs were fed the same (untreated) starter diet for 21 days until the end of experiment on day 35 (Table 1). During the study, all pigs had *ad libitum* access to feed and water. Starting at weaning BW was measured weekly, whereas feed intake was recorded daily until day 13 and weekly from day 15 to 35.

**Table 1:** Composition of the prestarter and the starter diets, % as fed basis, unless otherwise indicated

	Prestarter	Starter
**Ingredient**		
Corn	34.9	35.0
Wheat	11.0	22.6
Barley	8.0	9.5
Extruded soybeans	14.6	5.0
Soybean meal (56 % CP)	4.2	-
Soybean meal (47 % CP)	-	13.7
Sweet milk whey powder	12.6	2.94
Fishmeal	7.0	5.0
Soybean oil	4.0	-
Lard	-	2.06
Trace elements and vitamin premix^1^	1.55	1.52
Calcium carbonate	0.82	0.50
Monocalcium phosphate	0.64	1.40
Salt	-	0.34
L-Lysine-HCl	0.36	0.32
DL-Methionine	0.16	0.05
L-Threonine	0.13	0.04
L-Tryptophan	0.04	0.03
**Calculated nutrient composition**		
Crude protein	19.4	18.7
Digestible amino acids^2^		
Lysine	1.24	1.14
Methionine	0.49	0.37
Methionine + cysteine	0.72	0.62
Threonine	0.76	0.64
Tryptophan	0.23	0.22
Digestible energy (MJ/kg)	14.9	14.4
Net energy (MJ/kg)	11.2	10.3

^1^Containing the following: vitamin A, 10,000 UI; vitamin D_3_, 2000 UI; vitamin E (alfa-tocopherol), 25 mg; vitamin B_1_, 1.5 mg; vitamin B_2_, 3.5 mg; vitamin B_6_, 2.4 mg; vitamin B_12_, 20 μg; vitamin K_3_, 1.5 mg; calcium panthotenate, 14 mg; nicotinic acid, 20 mg; folic acid, 0.5 mg; biotin, 50 μg; iron sulfate, 120 mg; calcium iodate, 0.75 mg; cobalt carbonate, 0.6 mg; copper sulfate, 150 mg; manganesium oxide, 60 mg; zinc oxide, 110 mg; sodium selenite, 0.37 mg; amoxycilin trihidrate 10 %, 300 mg; colistin sulphate 4 %, 80 mg; zinc oxide, 2^.^610 mg

^2^Ileal standardized digestibility

### Plasma collection and analysis

Blood samples were obtained from six randomly-chosen pigs per treatment via jugular venipuncture on day 1, 7, and 14 after 12 h of feed deprivation. Samples were collected into tubes containing EDTA and aprotinin (BD Vacutainer®), held in ice-cold water for 30 min, centrifuged at 2000 × g for 10 min, stored at −80 °C, and analyzed later on for bioactive GLP-2 by radioimmunoassay as described previously [19].

### Tissue collection

On day 15 after 3 h of feed deprivation, 6 pigs per treatment were euthanized with an intravenous injection of sodium pentobarbital (200 mg per kg of BW; Fatro Ibérica, Spain). The abdomen was opened and the intestines were removed and dissected into sections arbitrary designated as jejunum (from the pyloric sphincter to the first Peyer’s patch), ileum (from the first Peyer’s patch to the ileocecal valve) and large intestine (from the ileocecal valve to the rectum). Intestinal sections were measured, flushed with saline, and weighted. A 10-cm segment was removed from the midsection of the jejunum and ileum, divided into 5-cm halves, and opened longitudinally. Half of these samples were fixed in 10 % buffered formalin for subsequent histological examination, whereas mucosal scrapings were taken from the other half and stored in RNA*later*® (Ambion, USA) at −80 °C until analysis of gene expression.

### Morphometric analysis

Samples of jejunum and ileum were dehydrated and embedded in paraffin, sectioned (~4 *μ*m), and stained with hematoxylin and eosin. Villus height, crypt depth, number of intraepithelial lymphocytes in villi, and number of goblet cells in crypts were measured in 10 well-oriented villi and crypts using a light microscope (BHS, Olympus) and a linear ocular micrometer (Olympus, Microplanet). All determinations were done by the same person, who was blinded to treatments, at 400× magnification as described previously [20].

### Real-time RT-qPCR analysis

Total RNA from intestinal mucosal scrapings was extracted and first strand cDNA synthesized as previously described [16]. Approximately 2 μg of RNA with an average A260/A280 of 1.9 were retrotranscribed. Primers and optimal PCR conditions for porcine *interleukin-10 (IL-10), tumor necrosis factor alpha (TNF-α), glucagon-like peptide-2 receptor (GLP-2R), proglucagon (GCG), sodium-dependent bile acid transporter (ASBT), tata box-binding protein (TBP), beta actin (ACTB)* [16], *zonula occludens-1 (ZON-1), occludin (OCLN)* [21] *epidermal growth factor receptor (EGFR)* [22] and *interleukin-6* (*IL-6*) [23] were taken from literature. These genes were previously shown to play a role in mediating the effects of bile acids on gut mucosal inflammation and barrier function [16–18]. Gene expression was determined in the jejunum and ileum, except for *EGFR* and *IL-6* that were examined only in ileal samples. All samples were run in triplicate in an ABI Prism 7300 Sequence Detector System (Applied Biosystems) using SYBR Green Master Mix (Applied Biosystems) and specific primers for each gene, as previously described [16].

### Statistical analysis

Analyses were performed using the mixed-model procedure of SAS (release 9.2, SAS Institute Inc.). Performance data (BW, average daily gain, feed intake and feed conversion) for animals that were slaughtered on day 15 (*n* = 6) and those that completed the study on d 35 (*n* = 12) were analyzed separately. These results and GLP-2 data were analyzed using a mixed-effect model with repeated measures in which pig within treatment was used as random variable whereas treatment, time (day or week), and the interaction treatment by time were considered fixed. The smallest value for the Akaike’s information criterion was used to identify the most appropriate covariance structure. The same model but without repeated measures was used to analyze intestinal weight, length, and morphology. To achieve normality, data for GLP-2 were transformed prior to analysis. Least squares means were separated into significant effects using the Fisher adjustment option of SAS. Differences in gene expression resulting from the comparison of the CDCd group with the CONd group were determined using a linear mixed-model in which treatment was included as fixed effect and the sample as random [24]. Gene specific residual variance (heterogeneous residual) was fitted to the gene effect [25]. For genes displaying efficiencies different from 2 (E ≠ 2), Ct values were adjusted according to the model described by Steibel et al. [24]. The geometric mean of the reference genes *TBP* and *ACTB* was used to correct Ct values of target genes [26]. Differences among treatments were considered to be significant when *P* < 0.05, whereas when *P* > 0.05 but < 0.10 differences were considered to indicate a trend towards a significant effect.

## Results

### Animal performance

**Fig. 1:**
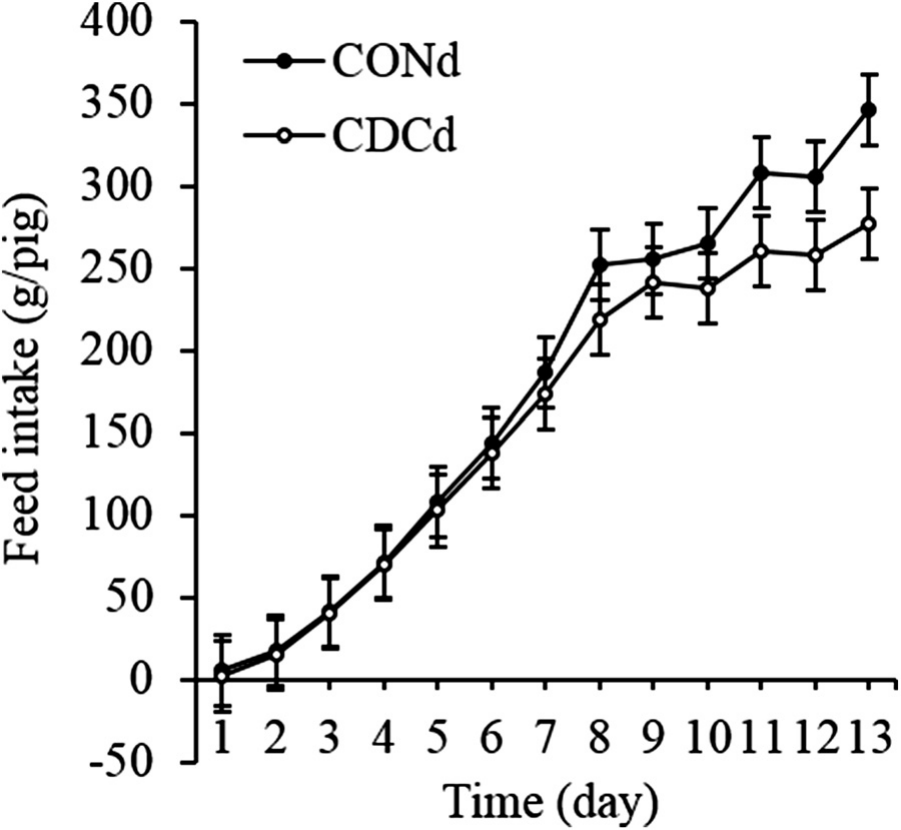
Accumulated feed intake (g/pig) from day 1 to 13 postweaning of piglets fed CONd or CDCd prestarter diet. Values are least squares means ± SEM, *n* = 18 per treatment. CONd, prestarter diet; CDCd, prestarter diet supplemented with 60 mg of chenodeoxycholic acid per kg of initial BW

**Fig. 2:**
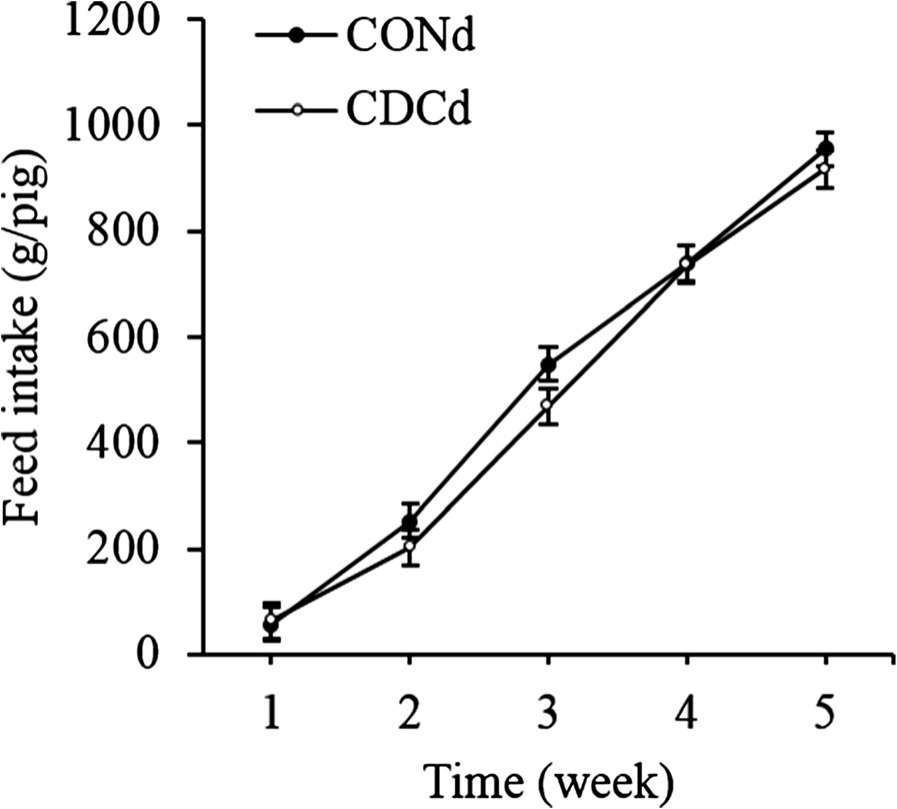
Accumulated feed intake (g/pig) from week 1 to 5 postweaning of piglets fed CONd or CDCd prestarter diet. Values are least squares means ± SEM, *n* = 12 per treatment. CONd, prestarter diet; CDCd, prestarter diet supplemented with 60 mg of chenodeoxycholic acid per kg of initial BW

The onset of feed consumption after weaning and its time course during both the first 13 days of exposure to treatments (Fig. 1) and the 5 weeks of study (Fig. 2) were similar between treatment groups. Likewise, supplementing the CONd prestarter diet with CDC did not alter weight gain of piglets that ended the study either on days 15 or 35 (Table 2). In both treatment groups the incidence of diarrhea was low and did not differ between them (Table 2). In addition, animals exhibited normal behavior and signs of adverse treatment effects were not observed during the study.

**Table 2:** Performance of piglets fed CONd or CDCd prestarter diet^a^

	Treatments		SEM^b^	P > F
	CONd	CDCd		
**0 to 15 days of study**				
*n*	6	6		
BW d 0 (kg)	6.3	6.3	0.21	0.83
BW d 14 (kg)	8.4	8.5	0.21	0.39
Average daily gain (g/d)	150	160	13.9	0.63
Average daily feed intake (g/d)	211	214	10.0	0.81
**0 to 35 days of study**				
*n*	12	12		
BW d 0 (kg)	6.2	6.2	0.53	0.44
BW d 35 (kg)	18.5	17.6	0.53	0.44
Average daily gain (g/d)	352	325	21.7	0.38
Average daily feed intake (g/d)	527	479	24.6	0.18
Feed:Gain	1.45	1.21	0.19	0.40
Diarrhoea^c^ (n)	3	3	---	0.46

^a^Data are least squares means. CONd, prestarter diet; CDCd, prestarter diet supplemented with 60 mg chenodeoxycholic acid per kg of initial BW

^b^Pooled SEM

^c^Number of events (P > *χ* ^2^)

### Plasma GLP-2

**Fig. 3:**
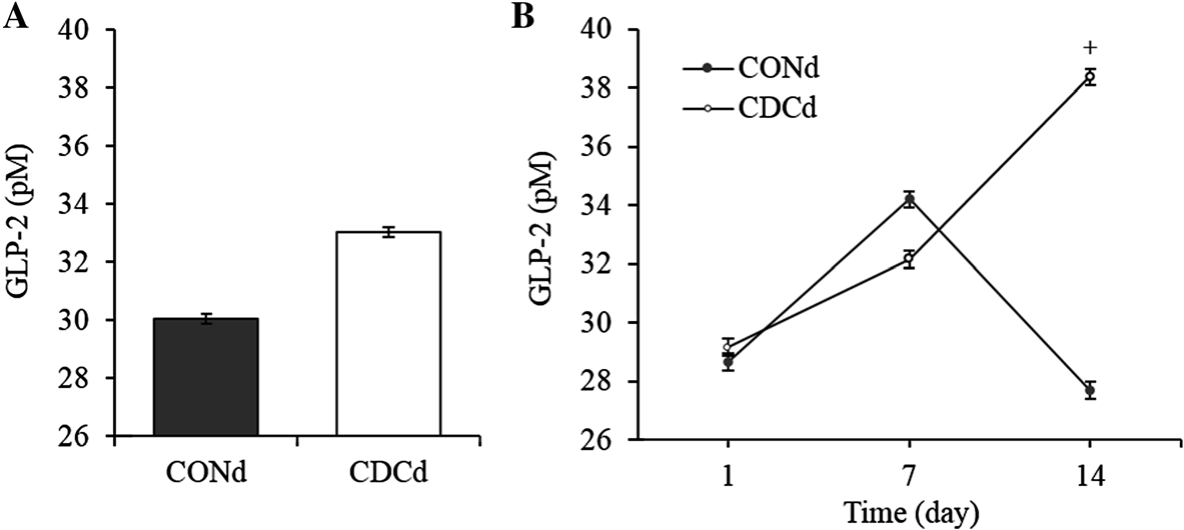
Changes in mean plasma (**A**) and circulating (**B**) GLP-2 concentration in piglets fed CONd or CDCd prestarter diet. Values are least squares means, *n* = 6 per treatment, A. SEM = 0.033. B. SEM = 0.056, ^+^ *P* = 0.07. CONd, prestarter diet; CDCd, prestarter diet supplemented with 60 mg of chenodeoxycholic acid per kg of initial BW

Although mean plasma GLP-2 did not differ between treatments (Fig. 3A), feeding CDCd tended (P < 0.07) to increase the concentration of circulating GLP-2 by 39 % on day 14 after 2 weeks of treatment exposure (Fig. 3B).

### Intestinal growth and morphology

The inclusion of CDC in the prestarter diet tended to enhance the weight of the large intestine (P < 0.10) but did not modify the size (weight and length) of the small intestine (Table 3). In addition, the morphology of the mucosa from the jejunum and ileum was similar between treatments, with the exemption of the ileal crypts that were deeper (P < 0.04) in pigs fed CDC (Table 4).

**Table 3:** Intestinal weight and length of piglets fed CONd or CDCd prestarter diet^a^

	Treatments		SEM	P > F
	CONd	CDCd		
**Organ weight (g/kg BW)**				
Duodenum + jejunum	32.8	34.7	1.32	0.33
Ileum	8.0	7.9	0.30	0.80
Small intestine	40.9	42.7	1.45	0.41
Large intestine	16.1	17.9	0.71	0.10
Whole intestine	57.0	60.5	1.64	0.16
**Organ length (cm/kg BW)**				
Duodenum + jejunum	100	102	4.8	0.83
Ileum	19.8	18.2	0.70	0.13
Small intestine	120	120	4.9	0.96

^a^Data are least squares means, *n* = 6. CONd, prestarter diet; CDCd, prestarter diet supplemented with 60 mg chenodeoxycholic acid per kg of initial body weight

**Table 4:** Intestinal morphology of piglets fed CONd or CDCd prestarter diet^a^

	Treatments		SEM	P > F
	CONd	CDCd		
**Jejunum**				
Villus height (μm)	454	419	23.8	0.31
Crypt depth (μm)	186	181	5.6	0.59
Villus:Crypt ratio	2.5	2.3	0.14	0.48
Intraepithelial lymphocytes (n/villus)	30.7	29.2	1.77	0.55
Goblet cells (n/villus)	6.8	7.0	0.44	0.70
**Ileum**				
Villus height (μm)	403	410	21.6	0.82
Crypt depth (μm)	133	153	6.0	0.04
Villus:Crypt ratio	3.1	2.7	0.16	0.13
Intraepithelial lymphocytes (n/villus)	27.7	30.3	1.60	0.27
Goblet cells (n/villus)	6.9	7.5	0.49	0.37

^a^Data are least squares means, *n* = 6. CONd, prestarter diet; CDCd, prestarter diet supplemented with 60 mg chenodeoxycholic acid per kg of initial body weight

### Intestinal gene expression

**Fig. 4:**
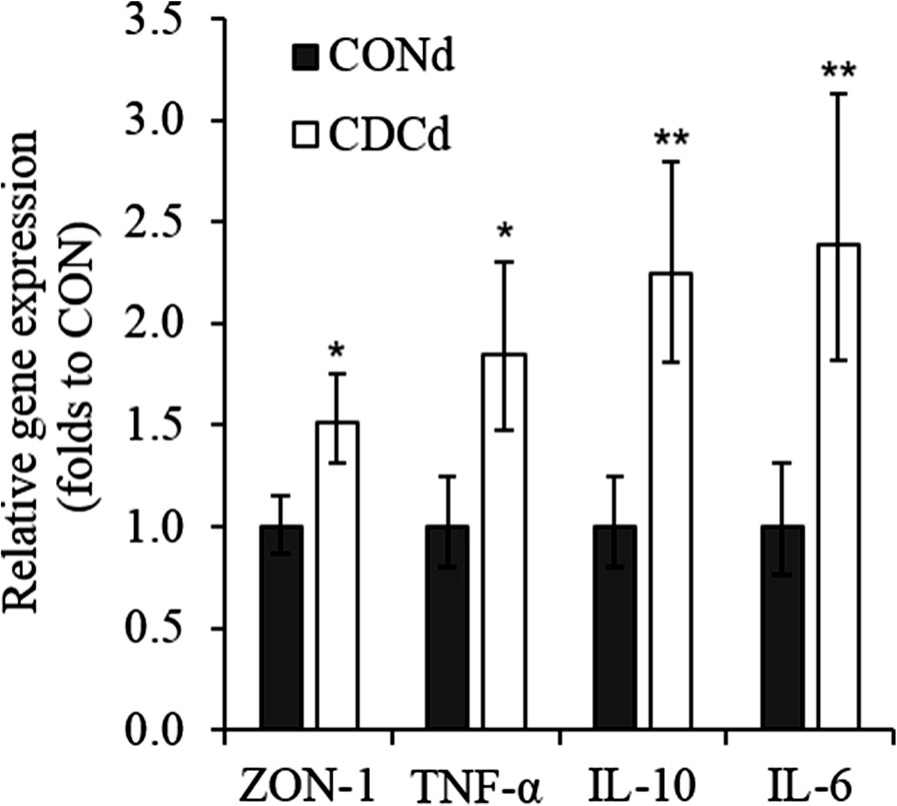
Relative abundance of ZON-1, TNF-α, IL-10 and IL-6 mRNA in the ileum of piglets fed CONd or CDCd prestarter diet. Relative gene expression values are fold change of the CDCd diet relative to the CONd which was set to be 1.0 (*n* = 6 per treatment). Bars indicate the 95 % confidence interval (Fold change up - Fold change low). CONd, prestarter diet; CDCd, prestarter diet supplemented with 60 mg of chenodeoxycholic acid per kg of initial BW; *ZON-1*, zonula occludens 1; *TNF-α*, tumor necrosis factor alpha; *IL-10*, interleukin 10; *IL-6,* interleukin 6. *P < 0.05, **P < 0.01

The feeding of CDCd during the 14 days that followed EW did not modify the relative concentration of mRNA transcripts from genes examined in the jejunal mucosa (data not shown). Although expression of *OCLN, GLP-2R, ASBT, EGFR* and *GCG* was similar between groups (data not shown), the expression of *ZON-1* (P < 0.02)*, TNF-α* (P < 0.02)*, IL-10* (P < 0.006), and *IL-6* (P < 0.01) increased 1.5, 1.9, 2.2 and 2.4 folds, respectively, in the ileum of CDCd-fed piglets relative to their CONd counterparts (Fig. 4).

## Discussion

Feeding piglets a cereal-based diet supplemented with CDC for 2 weeks after EW induced genes involved in the barrier function and protection of the intestinal mucosa with marginal effects on the concentration of circulating GLP-2 and mass of the intestine. The enteroprotective action of CDC was not associated with changes in food intake and BW gain either during the period of CDC supplementation or after withdrawal of CDC from the diet. The low incidence of diarrhea observed in both treatment groups suggests that keeping pigs in individual pens under high sanitary conditions might have hindered improvements in animal performance otherwise mediated by CDC. Taken together, data indicate that the oral administration of bile acids to weaned pigs has the potential to improve the protection of the intestinal mucosa independently of relevant changes in gut growth.

Weaning-induced intestinal atrophy and dysfunction are associated with a transient decrease in circulating GLP-2 [4, 5]. In a previous study, we have showed that the intragastric administration of a single dose of CDC to piglets during the first 6 days after EW remarkably increased the plasma concentration of endogenous GLP-2 but that this response tended to enhance only the length and weight of the ileum [16]. Thus, we speculated that supplementing the postweaning diet with CDC with the aim of distributing its enteral supply throughout the day may augment the nutrient-dependent secretion of GLP-2 and improve its efficacy to preserve gut integrity immediately after EW. We observed that plasma GLP-2 tended to increase at the end of the period of exposure to CDC on day 14, when food intake increased on average by 160 g/d (178 %) relative to the first postweaning week. Coincidentally, in our previous study piglets consumed during the first 6 days after EW about 66 % more feed (+48 g/d) than in the present study [16]. It seems therefore that there is a minimum of enteral nutrition required for CDC to potentiate the release of GLP-2 in animals fed solid diets. Although this effect was paralleled by a deepening of the ileal crypts, which is a distinctive trophic action of GLP-2 [5], only the mass of the large intestine was marginally increased. Paradoxically, enteral administration of CDC prevented gut atrophy in newborn piglets fed via parenteral [15]. In line with our findings, however, recent studies also observed modest enlargements of the epithelium of the intestine of enterally-fed weanling pigs in response to prolonged treatment with exogenous GLP-2 [11, 12]. Collectively, data indicate that the intestine of pigs remains responsive to the trophic effect of bile acids after weaning, an action presumably mediated by GLP-2, but that the magnitude of this effect is rather small and likely irrelevant from a developmental standpoint. However, one cannot rule out that the deepening of crypts induced by CDC might accelerate the recovery of the intestinal mucosa function after weaning and contribute to maintain TJ and adequate enterocyte turnover.

A critical function of the intestinal epithelium is to form a dynamic physical barrier to luminal contents to protect the host from infection and chronic exposure to inflammatory stimuli. Adjacent mucosal cells accomplish this by interacting through TJ proteins that are connected to the actin cytoskeleton and regulate the intestinal paracellular permeability [27]. Importantly, mounting evidence links increased gut permeability with intestinal inflammation, systemic immune activation, and disease progression in humans and animals [28]. Because EW dysregulates intestinal permeability in pigs [3, 29], targeting TJ proteins may illuminate ways to maintain the integrity of the intestinal barrier and thereby improve piglet health, growth, and welfare during the weaning period. Based on this prediction and the notion that bile acids [17, 18] and GLP-2 [30] regulate the expression and (or) cellular distribution of TJ proteins, we decided to examine the impact of dietary supplementation with CDC on the expression of some genes involved in the control of the barrier function of the intestinal mucosa. We found that feeding CDCd resulted in proinflammatory (i.e., *TNF-α* and *IL-6* expression) and anti-inflammatory (i.e., *IL-*10 expression) responses that were associated with increased concentration of *ZON-1* transcripts in the epithelium of the distal small intestine. Considering that during the development of intestinal inflammation TNF-α disrupts TJ [31] whereas IL-10 antagonizes its action [32], it seems reasonable to suggest that CDC triggered a homeostatic immune response that ultimately appeared to enhance the integrity of TJ of the intestinal epithelium. The question as to whether these effects were mediated directly by CDC via activation of the bile acid sensors TGR5 [18] and farnesoid X-activated receptor (FXR) [17] or indirectly via enhanced released of GLP-2 [30] cannot be addressed with data reported herein. However, the observation that the anti-inflammatory action of GLP-2 involves suppression of both crypt-cell proliferation and inflammatory cytokines through a mechanism unrelated to Th2 cytokines such as IL-10 [33], suggests that the tolerogenic response triggered by CDC was not associated with GLP-2. Yet, it is more important to note that in a recent study with early-weaned pigs weaning disrupted intestinal permeability partly by repressing the expression of TJ proteins, including ZON-1, and that this effect lasted for 14 days postweaning albeit the morphology of the intestinal mucosa was fully recovered by then [34]. Therefore, our findings support the proposal that the oral administration to pigs of bile acids, or compounds that mimic their action, holds potential for enhancing the integrity of the mucosal barrier at weaning and beyond this critical time.

It is widely accepted that disorders caused by EW, including increased susceptibility to diarrhea and growth retardation, mainly result from the transient absence of feed consumption that follows EW [1]. Expectably, supplementing the postweaning diet with CDC comprehended the risk of aggravating EW-induced anorexia because of reduced diet acceptability and (or) enhanced satiety mediated centrally by GLP-2 [35]. We found, however, that feeding CDCd did not affect the onset of feed intake following EW nor the amount of feed consumed during the 5-weeks study. Likewise, feed intake of weanling pigs was not affected when the plasma concentration of GLP-2 was increased via the intragastric infusion of CDC [16] or the administration of exogenous GLP-2 at a supraphysiological dose [11]. Despite the absence of anorectic effects, the aforementioned enteroprotective impact of feeding CDCd did not translate into improved animal performance (i.e., BW gain and incidence of diarrhea). As suggested before, the high sanitary conditions under which this study was conducted might have accounted for such results. Certainly, the efficacy of exogenous GLP-2 for improving gut integrity during EW was most evident when pigs developed severe diarrhea [12]. Furthermore, bile acid-mimicking compounds administered orally to mice suppressed intestinal inflammation and signs of diarrhea in models of chemically-induced colitis [18, 36]. Thus, available data provide a rationale for exploring the value of bile acids and compounds that mimic their action as dietary supplements to improve performance of pigs under situations of increased incidence of enteric disorders.

## Conclusions

Supplementing the diet of early-weaned pigs with CDC enhanced the expression of genes involved in the protection and barrier function of the mucosa of the distal small intestine. These effects, however, were only associated with a trend towards increased concentration of endogenous GLP-2 and intestinal growth. Even though dietary supplementation with CDC did not affect feed intake, the high sanitary conditions that prevailed in this study might have negated improvements in piglet performance resulting from the enteroprotective action of CDC. Results from this study warrant further research to examine the use of bile acids and compounds that mimic their action as dietary interventions to improve gut health and performance of pigs under situations of increased susceptibility to enteric inflammation and infection (e.g., poor environmental hygiene, increased microbial exposure, physical stress, etc.).

## Abbreviations

EW: Early weaning
GLP: Glucagon-like peptide
GPBAR1: TGR5, G-protein-coupled bile acid receptor 1
CDC: Chenodeoxycholic acid
BW: Body weight
CONd: Control standard prestarter diet
CDCd: Standard prestarter diet supplemented with 60 mg CDC per kg of body weight
IL-10: Interleukin-10
TNF-α: Tumor necrosis factor alpha
GLP-2R: Glucagon-like peptide-2 receptor
GCG: Proglucagon
ASBT: Sodium-dependent bile acid transporter
TBP: Tata box-binding protein
ACTB: Beta actin
ZON-1: Zonula occludens-1
OCLN: Occludin
EGFR: Epidermal growth factor receptor
IL-6: Interleukin 6
TJ: Tight junction
FXR: Farnesoid X-activated receptor
